# Supplementary material for: Identification of photoperiod-induced specific miRNAs in the adrenal glands of Sunite sheep (Ovis aries)
Source: Front Vet Sci. 2022 Jul 22;9:888207. doi: 10.3389/fvets.2022.888207 (PMC9354845; doi:10.3389/fvets.2022.888207)
Supplement: Supplementary file 1 [file Data_Sheet_1.ZIP › Supplementary file/miRDeep2 file.zip!/miRDeep2 file/Know_Structure/SP42A3/oar-mir-376b.pdf]

miRBase precursor : oar-mir-376b
Total read count : 409
oar-miR-376e-5p read count : 159
oar-miR-376b-5p read count : 159
oar-miR-376b-3p read count : 90
remaining reads : 1

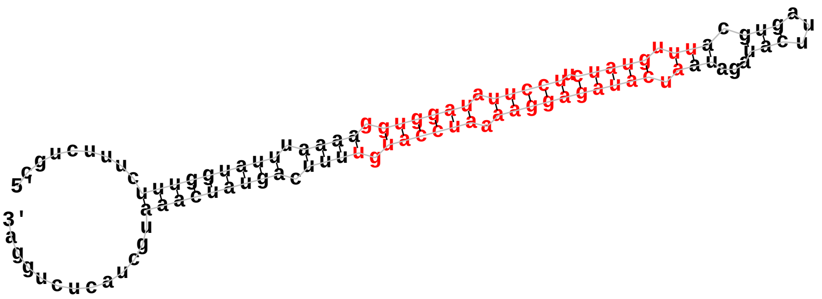

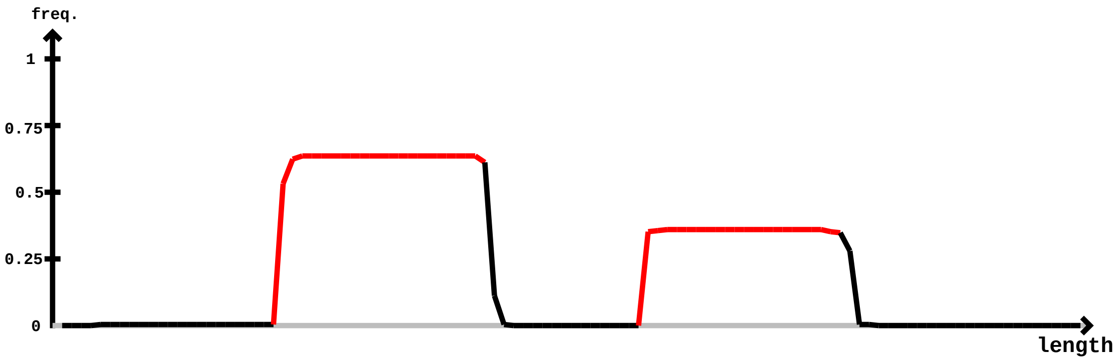


```
                                                                 oar-miR-376b-3p
                    oar-miR-376b-5p
                    oar-miR-376e-5p
5'- cgucuuucuuugguauuuaaaagguggauauuccuucuauguuuacgugauucauagauaaucauagaggaaaauccauguuuucaguaucaaaugcuacucugga   -3'    exp
    ........(((((((((.((((.((((((.(((((.(((((.(((.(((...)))...))).)))))))))).)))))).)))).)))))))))............    reads   mm   sample
    ...cuuucuuugguauuuaaaa....................................................................................    1       0    seq
    ......................gguggauauuccuucuauguu...............................................................    6       0    seq
    ......................gguggauauuccuucuauguuu..............................................................    110     0    seq
    ......................gguggauauuccuucuauguuua.............................................................    17      0    seq
    .......................guggauauuccuucuauguuu..............................................................    15      0    seq
    .......................guggauauuccuucuauguuua.............................................................    8       0    seq
    ........................uggauauuccuucuauguuua.............................................................    2       0    seq
    ........................uggauauuccuucuauguuuac............................................................    1       0    seq
    ............................................................aucauagaggaaaauccau...........................    2       0    seq
    ............................................................aucauagaggaaaauccaug..........................    1       0    seq
    ............................................................aucauagaggaaaauccaugu.........................    17      0    seq
    ............................................................aucauagaggaaaauccauguu........................    68      0    seq
    .............................................................ucauagaggaaaauccauguu........................    1       0    seq
    ..............................................................cauagaggaaaauccauguuuu......................    1       0    seq
```
